# Identification and validation of QTLs for tuber quality related traits in greater yam (*Dioscorea alata* L.)

**DOI:** 10.1038/s41598-022-12135-2

**Published:** 2022-05-19

**Authors:** Adou Emmanuel Ehounou, Fabien Cormier, Erick Maledon, Elie Nudol, Hélène Vignes, Marie Claire Gravillon, Assanvo Simon Pierre N’guetta, Pierre Mournet, Hâna Chaïr, Amani Michel Kouakou, Gemma Arnau

**Affiliations:** 1 CNRA, Station de Recherche sur les Cultures Vivrières (SRCV), 01 BP 633, Bouaké, Côte d’Ivoire; 2 UMR AGAP Institut, Univ Montpellier, CIRAD, INRAE, Institut Agro, 34398 Montpellier, France; 3 CIRAD, UMR AGAP Institut, 97170 Petit-Bourg, Guadeloupe France; 4 CIRAD, UMR AGAP Institut, 34398 Montpellier, France; 5 UFR Biosciences, Université Félix Houphouët Boigny, 22 BP 582, Abidjan 22, Côte d’Ivoire

**Keywords:** Biotechnology, Genetics, Plant sciences

## Abstract

Two *Dioscorea alata* populations were generated by hand pollination between contrasted diploid genitors. Population A (74F × Kabusa) was composed of 121 progenies while population B (74F × 14M) involved 193 progenies. These two populations were assessed over two consecutive years regarding important tuber quality traits. Analysis of variance showed that the genotype had the greatest influence on the phenotypic scores. Also for some traits, effect of the year_replicate was strong. The heritabilities of most traits were high. Based on these data and a reference high-density genetic map of greater yam, a total of 34 quantitative trait loci (QTLs) were detected on 8 of the 20 yam chromosomes. They corresponded to five of each of the following traits: tuber size, shape regularity, tubercular roots, skin texture, tuber flesh oxidation, six for oxidation ratio and three for flesh colour. The fraction of total phenotypic variance attributable to a single QTL ranged from 11.1 to 43.5%. We detected significant correlations between traits and QTL colocalizations that were consistent with these correlations. A majority of QTLs (62%) were found on linkage group LG16, indicating that this chromosome could play a major role in genetic control of the investigated traits. In addition, an inversion involving this chromosome was detected in the Kabusa male. Nine QTLs were validated on a diversity panel, including three for tuber size, three for shape regularity, two for skin texture and one for tubercular roots. The approximate physical localization of validated QTLs allowed the identification of various candidates genes. The validated QTLs should be useful for breeding programs using marker-assisted selection to improve yam tuber quality.

## Introduction

Yam belongs to the *Dioscorea* genus, which contains more than 600 species^1^, with *Dioscorea alata* and *D. rotundata* being the most cultivated forms^2^. World yam tuber production is estimated at more than 54 million t/year^3^. Around 95% of this production mainly comes from five West African countries, i.e. Nigeria, Benin, Togo, Ghana and Côte d'Ivoire, which constitute the yam belt. Yam is grown on all tropical continents, where it is a staple food for over 300 million people^4^. The *D. alata* yam species is vegetatively propagated^5^, but can also reproduce sexually from fertile genitors^6^. This species is also dioic and polyploid, with three levels of ploidy, i.e. 2n = 2x = 40 (diploid), 2n = 2x = 60 (triploid), 2n = 2x = 80 (tetraploid), with a base chromosome number of x = 20^7^. *D. alata* has a high yield potential, is vigorous, grows on all types of soil, multiplies easily (bulblets and seeds) and has a long conservation capacity, thus reducing postharvest loss^8^. Because of this high genetic potential of *D. alata* yam, it the most cultivated species^9^ and is also a major advantage in terms of food security. Several genetic improvement programs focused on this species are under way in the tropics which are mainly geared towards developing new varieties with high yields, good quality and good tolerance to diseases and pests^6,8,10–13^. However, yam variety selection is essentially based on phenotypic observations, which is often slow, difficult and burdensome. The use of molecular tools such as molecular markers could make it possible to select target traits more quickly. The development of these markers as selection tools is based on a reliable genetic map of the genome. Substantial progress has been recently made in genetic mapping of key traits using high-density genetic maps developed with next-generation sequencing technologies in many crops^14–16^.

In *D. alata* yam, genetic maps were first generated using dominant amplified fragment length polymorphism (AFLP) markers to identify resistance genes to anthracnose^17,18^. Recently, new genetic linkage maps of *D. alata* were obtained using codominant markers, i.e. EST-SSRs and SNPs^9,19,20^. Very few studies have been undertaken to search for QTLs related to morphological and agronomic characteristics involved in tuber quality. In addition, the genetic bases of the traits that determine tuber quality are unknown.

Indeed, tuber quality is a key criterion in the evaluation and selection of new varieties^21,22^. Several characteristics involved in quality variability are routinely and conventionally measured in selection programs (tuber shape, tuber appearance, tubercular roots, flesh colour and oxidation). Slightly elongated, smooth and undigitized (regular shape) yams are preferred by both consumers and producers. Indeed, oval, slightly elongated and undigitized shapes facilitate harvesting and storage (low risk of breaks and injuries that can be conducive to rotting during storage). The smooth appearance of the tuber and the absence of tubercular roots make peeling easier during meal preparation. The flesh colour is also important because consumers generally prefer white-fleshed varieties for pounded yam preparations^23^. Tuber flesh oxidation (browning) is caused by an enzyme that reacts with oxygen in the air when the tuber is cut. This is a real problem because it affects the tuber quality^24^. QTL mapping—an important tool for breeders—could be used to combine all of these traits to create elite varieties. Since in species such as potato, sweet potato, and cassava they are determined by quantitative effect genes (QTLs)^25–27^. Two studies have recently been published on *D. alata* that revealed some QTLs for four important quality related traits (dry matter, oxidative browning, tuber size and shape), but none of these QTLs have been validated to date^20,28^.

We recently developed a high-density reference genetic map of this species containing 1579 SNPs, with a total length of 2613.5 cM and average distance of 1.68 cM between flanking markers^19^. This was used to search for QTLs related to tuber quality traits. It could be used to identify a number of characteristics, including morphological and agronomic tuber traits. The aim of this study was to assess the genetic control of *D. alata* tuber quality traits and identify QTLs involved in their variability.

## Materials and methods

### Materials

The *D. alata* mapping populations used in this study were full-sibling progenies derived from crosses between diploids with contrasted characters^19^. The hybridizations were performed in the French West Indies (Guadeloupe) at the French agricultural research and international cooperation for Development (CIRAD). Population A included 121 hybrids and was derived from a cross between a female breeding line (74F) and a male landrace (Kabusa). Population B included 193 hybrids and was derived from a cross between the same female line (74F) and a male breeding line (14M). The 74F female produces long tubers with rough skin, yellow flesh and tubercular roots. Kabusa and 14M males, respectively produce cylindrical and oval tubers with smooth skin and white and white-cream coloured flesh, respectively. Kabusa has no tubercular roots whereas 14M has a few. Among the genitors, only the 74F female oxidizes.

A diversity panel of 28 *D. alata* varieties was used for QTL validation. This panel was used only to validate the detected QTLs with regard to the following four parameters: length/width ratio, tuber shape regularity, tubercular roots and skin texture. These varieties were planted together in the same plot at the Roujol experimental station (Guadeloupe) in 2018 and phenotyped in 2019.

### Experimental design

Both populations A and B were planted in the two consecutive years (2017 and 2018) at Roujol experimental station (16° 10′ 56″ N, 61° 35′ 24″ W, 10 a.s.l.). Each population was planted in two blocks on pre-made ridges. Each genotype was randomly distributed in each block and included a total of 18 repetitions (2 blocks × 9 plants). 100 g of tuber per repetition was used for planting. The distance between ridges was 1.5 m, and on each ridge plants were separated by 0.5 m for the same genotype, with 1 m between different genotypes.

### Phenotyping

Phenotyping of mapping populations along with their progenitors was performed in two consecutive years (2018–2019). Seven traits were measured: tuber size (length and width), tuber shape regularity, the presence of roots on the tuber surface (tubercular roots), skin texture (smooth or rough), flesh colour, tuber flesh oxidation (center of the tuber) and the proportion of tuber oxidization. The observations of the first four traits were made on all tubers of each genotype. While the other traits were measured on four tubers randomly selected from each block.

The size of each tuber was quantitatively measured according to the length (longest part of tubers) and width (widest part of tubers).The tuber flesh colour was monitored and noted immediately after cutting the tuber longitudinally. Tuber flesh oxidation (center of tubers) was noted by visual observation 15 min after cutting a fresh tuber. The proportion of tuber oxidization was measured with a meter tape 15 min after cutting tubers longitudinally. Phenotyping of the diversity panel was performed as for biparental segregation populations. All measured parameters and their modalities are shown in Table 1.

**Table 1:** List of parameters measured on populations A and B and parents during the years 2018 and 2019.

Parameters	Modalities
Size (ratio L/W)	L/W (Length (L) and Width (W))
Regularity of tuber shape	High = 0; low = 1; absence = 2
Tubercular roots	Absence = 0; few = 1; a lot = 2
Skin texture	Smooth = 0; rough = 1
Flesh colour of fresh tuber (center)	White = 1; other white = 2; others colours = 3
Flesh oxidation (center of tuber)	No = 0; yes = 1
Oxidation ratio	L0 = length of oxidation 15 min after cutting the fresh tuber

### Statistical analysis of phenotypic data

Statistical analyses were performed using XLSTAT version 19.03.44616 and with R. ANOVA and heritability analysis were computed in R ^29^. Variance estimates were used to estimate broad–sense heritability according to the formula of Domanski et al.^30^: H^2^ = σ2 g/(σ2 g + σ2 ge + σ2 e), where σ2g, σ2ge and σ2e are the variance components for genotypes, genotypes × year_replicate and residual variation. This heritability is the ratio of genetic to total variance. The error variance was divided by the appropriate number of replicates each year and averaged over years to give an overall error variance. For traits deviating from normality, several tranformations (ln, square root …) were tested. The least-skewed transformed data were used to extract the Best Linear Unbiased Predictors (BLUP) for each data^31^. BLUPs values were used for correlation estimation and QTL detection. As several traits did not follow a normal distribution, phenotypic correlations among traits were calculated using the non-parametric Spearman correlation coefficient.

Histograms, box-plots and Spearman correlation tests were performed using XLSTAT. The different trait distributions were tested for normality using Shapiro-Wilk^32^ (1965) and JarqueBera tests^33^.

### Linkage maps

New LG16 linkage maps were constructed for each parent for detailed analysis of the structure of this chromosome using JoinMap 4.1 software^34^. These maps were generated using a larger number of progenies than those used to develop the reference genetic map. Indeed, when the reference genetic map was constructed, several progenies from populations A and B presented a missing data rate exceeding the set threshold (< 40% per individual) and could not be included in the analysis^19^. We therefore reperformed the GBS analyses for these progenies. SNP calling and filtering were performed as previously described^19^. Parental maps were generated from 597 segregating SNPs in population A and 404 segregating SNPs in population B. Maps were obtained using recombination frequencies below 0.45, a LOD score of 10, the regression algorithm with two ordering rounds and the Kosambi mapping function.

### QTL mapping

The QTL analyses were performed on the previously published reference genetic map and generated from these populations. This map spanned 2613.5 cM and contained 1579 SNP markers^19^. The two population phenotyping datasets obtained for the two years combined with the *D. alata* reference map were used to identify regions associated with different tuber quality traits. QTL analysis was performed for each population separately on the best linear unbiased prediction (BLUP) values of clone effects using Map QTL version 6^35^. QTLs were initially detected using the interval mapping (IM) approach. Then the loci with the highest LOD scores were used as cofactors in analyses using the composite interval mapping (MQM) model to detect additional QTLs that might be masked by major QTLs. Confidence intervals of QTL positions were determined as two-LOD support intervals. Significance LOD score thresholds were calculated through permutation of 1000 iterations at the 0.05 probability level of significance.

### QTL validation

QTL validation was performed on a diversity panel of 28 varieties. SNP genotyping data for these varieties was generated by Sharif et al.^36^. A simple linear model that associates each phenotype with a SNP was tested using the Pearson correlation test and a 5% significance level. The Pearson R^2^ coefficient of determination and the p-value (Pearson) of each SNP marker thus were determined. All SNPs included in the QTL confidence intervals were tested including those outside the closest ones.

In addition, an ANOVA analysis was performed for each of the validated SNP markers to analyses whether the observed genotypic classes were significantly different at p < 0.05.

### Permission statement

All the experimental methods including field studies were performed in accordance with relevant guidelines and regulations.

## Results

### Phenotyping

**Figure 1:**
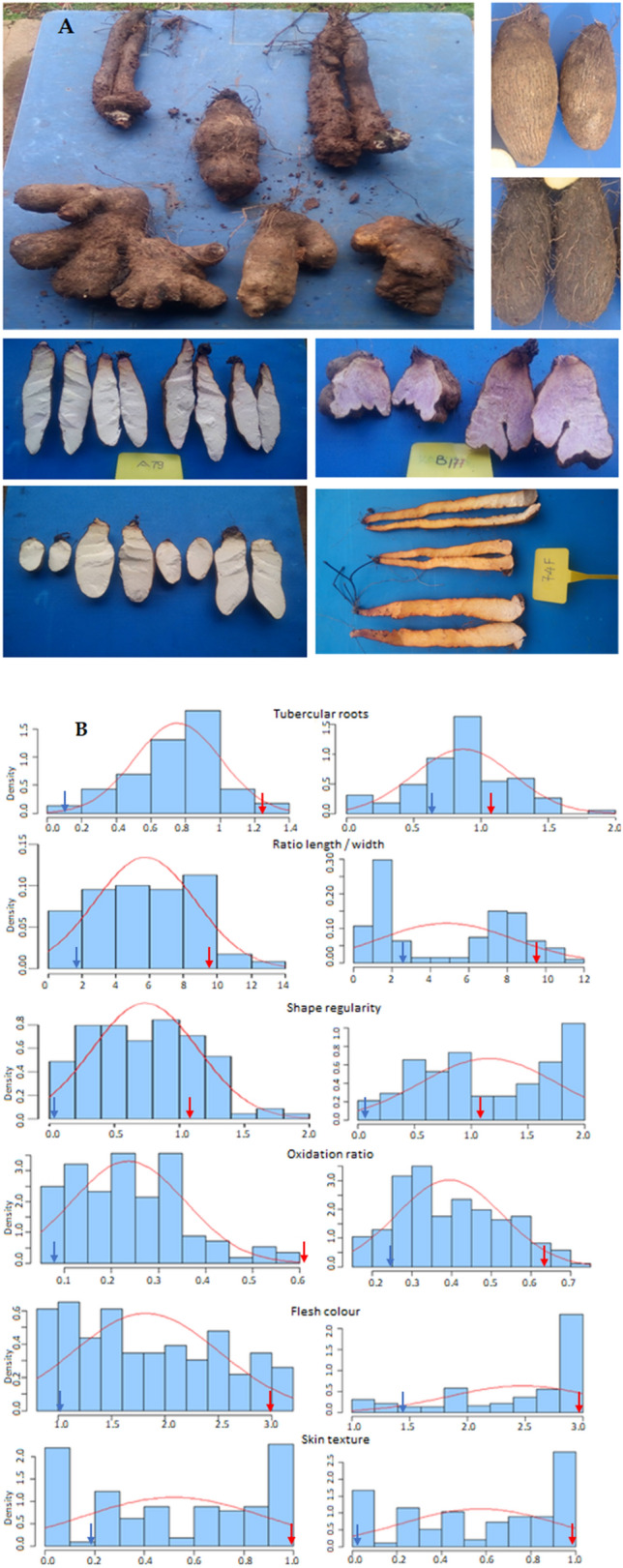
(**A**) Phenotypic variation for tuber shape, shape regularity, tubercular roots and flesh colour traits. (**B**) Phenotypic distribution of quality traits in population A (74F × Kabusa, on the left) and population B (74F × 14M, on the right).

A large variability was observed in both progenies for different evaluated traits (Fig. 1, Table 2). The analysis of variance in mapping population A (74F × Kabusa) demonstrated significant effects of genotype, year_replicate on ratio length/width, tubercular roots, skin texture and flesh colour (Table 2). For the mapping population B (74F × 14M), there were significant genotype and year_replicate effects for all traits. The range of scores obtained for tubercular roots and shape regularity in progeny B was wider than in progeny A (Fig. 1). There was continuous variation in all of the studied traits, which is typical of quantitative and polygenic inheritance (Fig. 1). In population A, a normal distribution was confirmed for the length/width ratio, shape regularity, tubercular roots and oxidation ratio (p = 0.05). The distribution for skin texture and flesh oxidation deviated significantly from normality. Distributions in population B were generally skewed and/or significantly deviated from normality (p = 0.05, Fig. 1). Transgressive segregation was observed for many traits, with values being lower or higher than those of the parents. This phenomenon was particularly pronounced with regard to the higher values obtained for tubercular roots and irregular tuber shape in population B.

**Table 2:** Analysis of variance of ratio length/width, tubercular roots, skin texture, shape regularity, flesh colour, flesh oxidation and oxidation ratio.

Source	POPA					POPB				
	df	Mean Sq	F	*p*	h^2^	df	Mean Sq	F	*p*	h^2^
**Ratio L/W**										
Clone	111	30.90	12.04	< *0.0001*	0.92	180	38.25	13.81	< *0.0001*	0.94
Year_rep	3	14.91	5.81	*0.0007*		3	42.88	15.48	*0.0001*	
**Tubercular roots**										
Clone	111	0.21	1.48	*0.0052*	0.34	180	0.33	1.84	< *0.0001*	0.51
Year_rep	3	2.06	14.38	< *0.0001*		3	6.47	36.04	< *0.0001*	
**Skin**										
Clone	111	0.32	3.34	< *0.0001*	0.72	180	0.36	3.81	< *0.0001*	0.78
Year_rep	3	6.34	21.73	< *0.0001*		3	5.39	57.65	< *0.0001*	
**Regularity of tuber shape**										
Clone	111	0.51	2.02	< *0.0001*	0.55	178	1.07	4.55	< *0.0001*	0.82
Year_rep	3	0.35	1.37	0.2505 n.s		3	7.57	32.09	< *0.0001*	
**Flesh colour**										
Clone	111	5.30	17.57	< *0.0001*	0.96	175	3.56	16.04	< *0.0001*	0.96
Year_rep	3	23.77	78.80	< *0.0001*		3	14.66	66.09	< *0.0001*	
**Flesh oxidation**										
Clone	111	0.45	6.42	< *0.0001*	0.88	170	1.10	9.40	< *0.0001*	0.93
Year_rep	3	0.02	0.26	0.854 n.s		3	2.16	18.41	< *0.0001*	
**Oxidation ratio**										
Clone	110	0.07	9.40	< *0.0001*	0.91	159	0.11	6.42	< *0.0001*	0.87
Year_rep	3	0.10	6.04	*0.0004*		3	0.044	2.49	0.056 n.s	

Significant values are in [italics].

The broad sense heritabilities in population A were high for the length/width ratio, skin texture, flesh colour flesh oxidation and oxidation ratio traits (0.92, 0.72, 0.96 and 0.88 and 0,91), low and moderate for tubercular roots and tuber shape regularity traits (0.34 and 0.55), respectively. In population B, apart from the tubercular roots, all other characteristics had high heritabilities. Those obtained for tuber shape regularity and tubercular roots were significantly higher in population B (0.82 and 0.51) than in population A (0.55 and 0.34).

Several significant correlations were detected between analyzed traits in both populations. In population A, the flesh colour was positively correlated with the skin texture and flesh oxidation. The skin texture was positively correlated with the length/width ratio and flesh oxidation (Table 3). From population B, the flesh colour and length/width ratio were strongly and positively correlated with the skin texture, flesh oxidation and oxidation ratio, but negatively correlated with tubercular roots and tuber shape regularity. The skin texture was negatively correlated with tubercular roots and tuber shape regularity. The tuber shape regularity was negatively correlated with flesh oxidation. The flesh oxidation and the oxidation ration were strongly positively correlated (Table 4).

**Table 3:** Correlation coefficients (Spearman’s r values) computed mean values over years between ratio length/width, regularity of tuber shape, skin texture, tubercular roots, flesh colour, flesh oxidation, oxidation ratio of mapping population A (74F × Kabusa).

Traits	Flesh colour	Ratio L/W	Skin texture	Tubercular roots	Regularity tuber shape	Flesh oxidation	Oxidation ratio
Flesh colour							
Ratio length/width	0.423 n.s						
Skin texture	0.586*	0.694*					
Tubercular roots	0.028 n.s	− 0.058 n.s	0.137 n.s				
Regularity tuber shape	0.378 n.s	0.089 n.s	0.410 n.s	0.169 n.s			
Flesh oxidation	0.509*	0.411n.s	0.417*	0.003 n.s	0.135 n.s		
Oxidation ratio	0.113 n.s	0.135 n.s	− 0.067 n.s	− 0.076 n.s	− 0.186 n.s	0.284 n.s	

Significant values are in [bold].

n.s: not significant.

*Significant at p < 0.05.

**Table 4:** Correlation coefficients (Spearman’s r values) computed mean values over years between ratio length/width, regularity of tuber shape, skin texture, tubercular roots, flesh colour, flesh oxidation and oxidation ratio of mapping population B (74F × 14M).

Traits	Flesh colour	Ratio L/W	Skin texture	Tubercular roots	Regularity tuber shape	Flesh oxidation	Oxidation ratio
Flesh colour							
Ratio length/width	0.615*						
Skin texture	0.590***	0.670**					
Tubercular roots	− 0.332***	− 0.6241*	− 0.312*				
Regularity tuber shape	− 0.503***	− 0.799***	− 0.633**	0.603***			
Flesh oxidation	0.644***	0.625*	0.561*	− 0.296*	− 0.527*		
Oxidation ratio	0.556***	0.621*	0.590*	− 0.334*	− 0.569*	0.761***	

Significant values are in [bold].

n.s: not significant.

*Significant at p < 0.05, **significant at p < 0.01 ***significant at p < 0.0001.

### Linkage map

**Figure 2:**
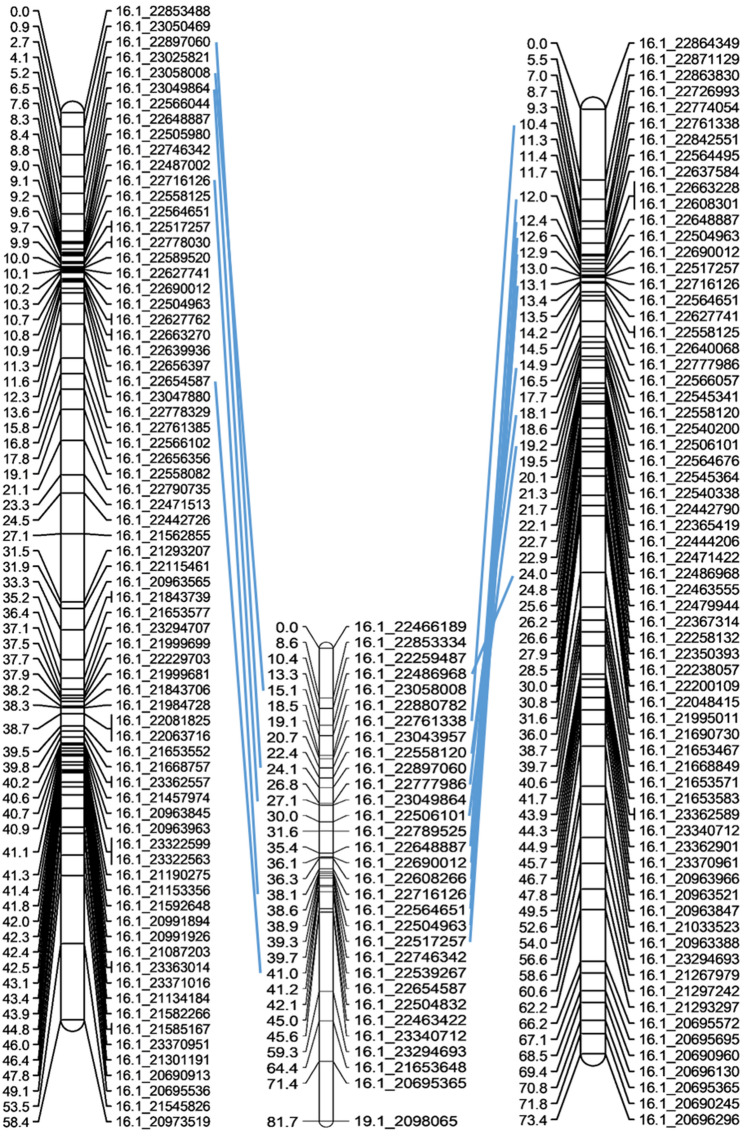
New parental maps of chromosome LG16 for the male Kabusa (on the left) and female 74F (on the right). The consensus map is in the middle. In blue are showed the common SNPs.

New chromosome linkage maps were constructed for each parent for detailed analysis of the structure of chromosome LG16. The parental maps generated from population B revealed the presence of an inversion in the Kabusa male. Indeed, the marker order was inversed when comparing the Kabusa male and the 74F female for markers mapped between 10.5 and 41 cM on the reference map (Fig. 2). In parental maps generated from population B data (not shown), the marker order corresponded to that observed in the female parent. This led us to conclude that it was the genitor Kabusa which presented the inversion. In addition, the new map of the 14 M male (not shown) led to identification of the putative centromeric region (between 5 and 19 Mb) based on a comparison of recombination events versus physical positions on the *D. rotundata* pseudo-chromosome.

### QTL analysis

The QTL analysis results are presented in Table 5. Significant QTLs were found in both populations for the length/width ratio, skin texture, flesh colour, flesh oxidation and oxidation ratio, while they were only detected in population B for tuber shape regularity and tubercular roots. The LOD scores ranged from 3.5 to 16.72 and the corresponding phenotypic variance attributable to QTLs (R^2^) ranged from 11.1 to 43.5%. The majority of markers associated with QTLs were transmitted by both parents in population B, whereas they were just transmitted by the male or female parent in population A.

**Table 5:** QTL detected for mean (2018–2019) ratio length/width, skin texture, tubercular roots, shape regularity, flesh colour, flesh oxidation and oxidation ratio in the mapping populations A and B.

Trait	LG	Marker associated	Marker origin	QTL position	LOD	R^2^ (%)	Interval (%)
POPA							
Ratio L/W	LG16	16.1_2289706	M	25.1	4.51	17.5	0–32.6
	LG16	16.1_22654587	D	41.1	4.85	18.7	38.8–44.9
	LG15	15.1_6269438	D	80.0	3.61	14.3	71.6–87.7
	LG02	02.1_26794334	M	71.2	3.60	13.7	67.7–79.9
Skin texture	LG16	16.1_22761338	F	18.5	4.12	16.1	9.5–32.6
	LG16	16.1_22463422	D	44.1	5.88	22.2	39.7–44.9
	LG15	15.1_7629197	M	81.9	4.31	16.8	75.6–83.9
Flesh colour	LG16	16.1_23058008	M	16.1	5.24	20	4–28.1
	LG16	16.1_22539267	F	41.0	4.94	19	38.8–44.9
Flesh oxidation	LG16	16.1_22486968	F	14.3	5.68	21.5	6–22.3
	LG16	16.1_22648887	D	35.4	5.02	19.3	31.6–41.1
	LG15	15.1_8934406	M	123	4.72	17.5	122–125
	LG19	19.1_9513064	F	70.1	3.5	13.9	62.1–73.9
Oxidation ratio	LG16	16.1_22558120	F	22.4	12.28	40.8	12.4–70.4
	LG07	15.1_2205278	M	78.7	4.2	16.4	74.8–82.2
	LG06_M	06.1_31811266	M	57.8	3.57	14.1	51.5–62.3

POPB							
Ratio L/W	LG16	16.1_23043957	D	21.1	5.67	18.5	16.3–24.3
	LG16	16.1_22504963	D	38.9	6.23	20.1	38.8–49.5
	LG16	16.1_21653648	D	64.4	8.99	27.6	56.5–81.6
Regularity of tuber shape	LG16	16.1_23043957	D	20.7	6.25	19	18.5–24.1
	LG16	16.1_22504963	D	38.8	5.2	16	38.5–44.0
	LG16	16.1_21653648	D	64.4	11.37	32	58–67.4
	LG02	02.1_25284501	D	62.4	3.91	12.2	58.4–64.4
	LG01	01.1_400095	D	91.7	3.64	11.5	82.8–98.9
Tubercular roots	LG16	16.1_23043957	D	21.1	7.94	23.1	16.3–25.4
	LG16	16.1_22504963	D	38.8	7.77	22.7	38.8–45.5
	LG16	16.1_21653648	D	63.5	6.91	20.5	56.5–64.4
	LG19	21.1_1121998	D	163.1	4.39	13.5	156.8–163.1
	LG06_M	06.1_29183630	M	13.1	3.58	11.1	5.8–15.4
Skin texture	LG16	16.1_23043957	D	21.1	6.42	19.1	17.3–24.4
	LG16	16.1_22504963	D	38.8	5.7	18	38.0–44.1
	LG16	16.1_21653648	D	64.4	17.38	43.5	57.5–66.4
	LG17	17.1_7523202	M	86.3	4.25	13	82.6–94.5
Flesh colour	LG16	16.1_23043957	D	21.1	11.08	29.9	15.3–24.4
	LG16	16.1_22504963	D	38.9	9.36	28.1	38.5–49.0
	LG16	16.1_21653648	D	65.4	16.02	40	57.5–69.4
Flesh oxidation	LG16	16.1_23043957	D	21.1	5.7	18	15.3–24.4
	LG16	16.1_22504963	D	38.8	4.1	13.3	38.8–49.5
	LG16	16.1_21653648	D	64.4	7.04	21.1	55.5–81.6
Oxidation ratio	LG16	16.1_22558120	D	21.7	5.66	18.4	16.1–25.1
	LG16	16.1_22504963	D	38.8	6.23	20.1	38.5–39.7
	LG16	16.1_21653648	D	66.4	8.99	27.6	56.5–81.7
	LG07	07.1_1751053	D	103.1	3.67	12.4	102.8–107.8

*LG* linkage group, *Marker associated* closest marker to the highest LOD score, *M* inherited from male, *F* Inherited from female (74F), *D* inherited from both parents.

**Figure 3:**
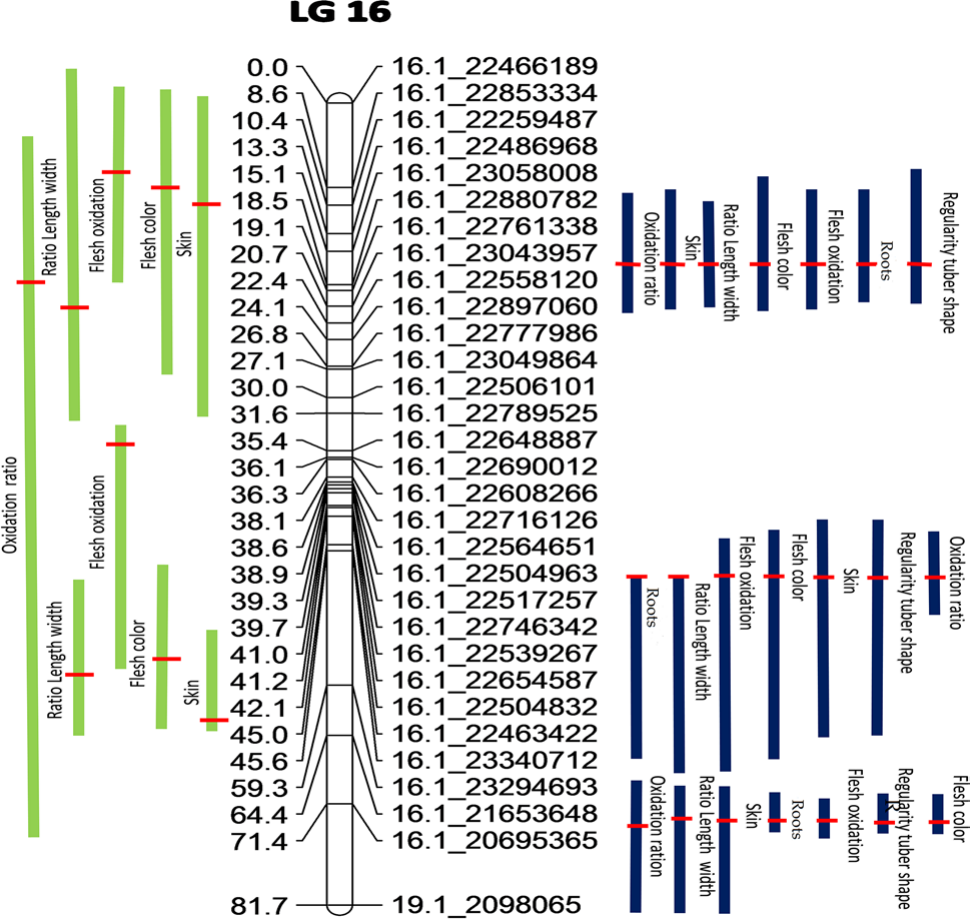
QTLs detected on the consensus map of chromosome LG16 for quality traits in population A (74F × Kabusa), in green and the population B (74F × 14M), in blue.

For the length/width ratio, four QTLs were identified in population A, two on chromosome LG16 (25.1 cM and 41.1 cM), one on LG15 (80 cM) and one on LG2 (71.2 cM). While in population B, three QTLs were identified on chromosome LG16 at positions 21.1 cM, 38.9 cM and 64.4 cM, with two 21.1 cM [16.3–24.3] and 38.9 cM [38.8–49.5] colocolizing with two in population A 25.1 cM [0–32.6] and 41.1 cM [38.8–44.9] (Fig. 3).

Five QTLs were detected for tubercular roots in population B on chromosomes LG06_M (13.1 cM), LG19 (163.1 cM) and LG16 (21.1 cM, 38.8 cM, 63.5 cM) explaining from 11.1 to 23.1% of total phenotypic variation.

Five QTLs were identified for tuber shape regularity in population B. Single QTLs were detected on chromosomes LG02 (62.4 cM) and LG01 (91.7 cM), while three QTLs were discovered (20.7 cM, 38.8 cM, 64.4 cM) on chromosome LG16. These QTLs explained 11.5 to 32% of the observed variation.

Three QTLs associated with the skin texture were revealed in population A, i.e.; one on chromosome LG15 and two on LG16. In population B, four QTLs affecting the skin texture were identified, i.e. three on chromosome LG16 and one on LG17.

A total of eight significant QTLs associated with flesh oxidative browning were detected on five different chromosomes (LG19, LG16, LG15, LG07 and LG06_M) explaining from 12.4 to 40.8% of the phenotypic variance.

Two and three QTLs were respectively detected for flesh colour in populations A and B, all located on chromosome LG16. The highest R^2^ value (40%) was found for the QTL located at position 65.4 cM.

Overlapping QTLs were detected on LG16 for many traits in both populations (Fig. 3). In addition, QTL intervals for length/width ratio and skin texture also overlapped on chromosome LG15. Non-similar phenomena were observed on other chromosomes containing more than one QTL (LG02, LG07, LG06_M and LG19).

### QTL validation

Table 6 presents SNP markers that showed a significant association with the phenotypic data from the validation panel.

**Table 6:** Summary of QTLs validated on the diversity panel.

Trait	SNPs	LG	Position (cM)	R^2^	P
Ratio length/width	16.1_23049864	LG16	27.096	0.202	0.016
	16.1_22716126	LG16	38.055	0.309	0.002
	16.1_22746342	LG16	39.742	0.227	0.01
	06.1_16775930	LG15	75.687	0.213	0.013
Regularity of tuber shape	16.1_22539267	LG16	41.045	0.154	0.047
	16.1_22506101	LG16	30.033	0.376	0.001
	02.1_19043957	LG02	55.857	0.178	0.032
Skin texture	16.1_22564651	LG16	38.598	0.169	0.041
	15.1_6502671	LG15	78.498	0.171	0.036
Tubercular roots	16.1_21653648	LG16	64.429	0.182	0.033

*LG* linkage group, *R*^*2*^ Pearson determination coefficients, *p* level of significance (p < 0.05).

Three of the five QTLs that were identified for the length/width ratio were validated on the diversity panel. Two significant SNPs were validated on chromosome LG16, i.e. one SNP 16.1_23049864 (R^2^ = 0.202, p < 0.016) was found at position 27.1 cM in the confidence interval of the QTL identified at position 25.1 cM [0–32.6] and the second SNP 16.1_22746342 (R^2^ = 0.227 p < 0.010) was found at position 39.742 cM in the confidence interval of the QTL identified in populations A [38.8–44.9] and B |38.8–49.5]. In addition, another SNP marker, i.e. 16.1_22716126 which had a stronger association with the phenotypic data (R^2^ = 0.309, p < 0.002), was found near these QTLs at position 38.055 cM. Finally, a significant SNP, i.e. 06.1_16775930 (R^2^ = 0.213, p < 0.013), was found at position 75.687 cM in the confidence interval of the QTL which was identified in population A at position 80.0 |71.6–87.7] on chromosome LG15.

**Figure 4:**
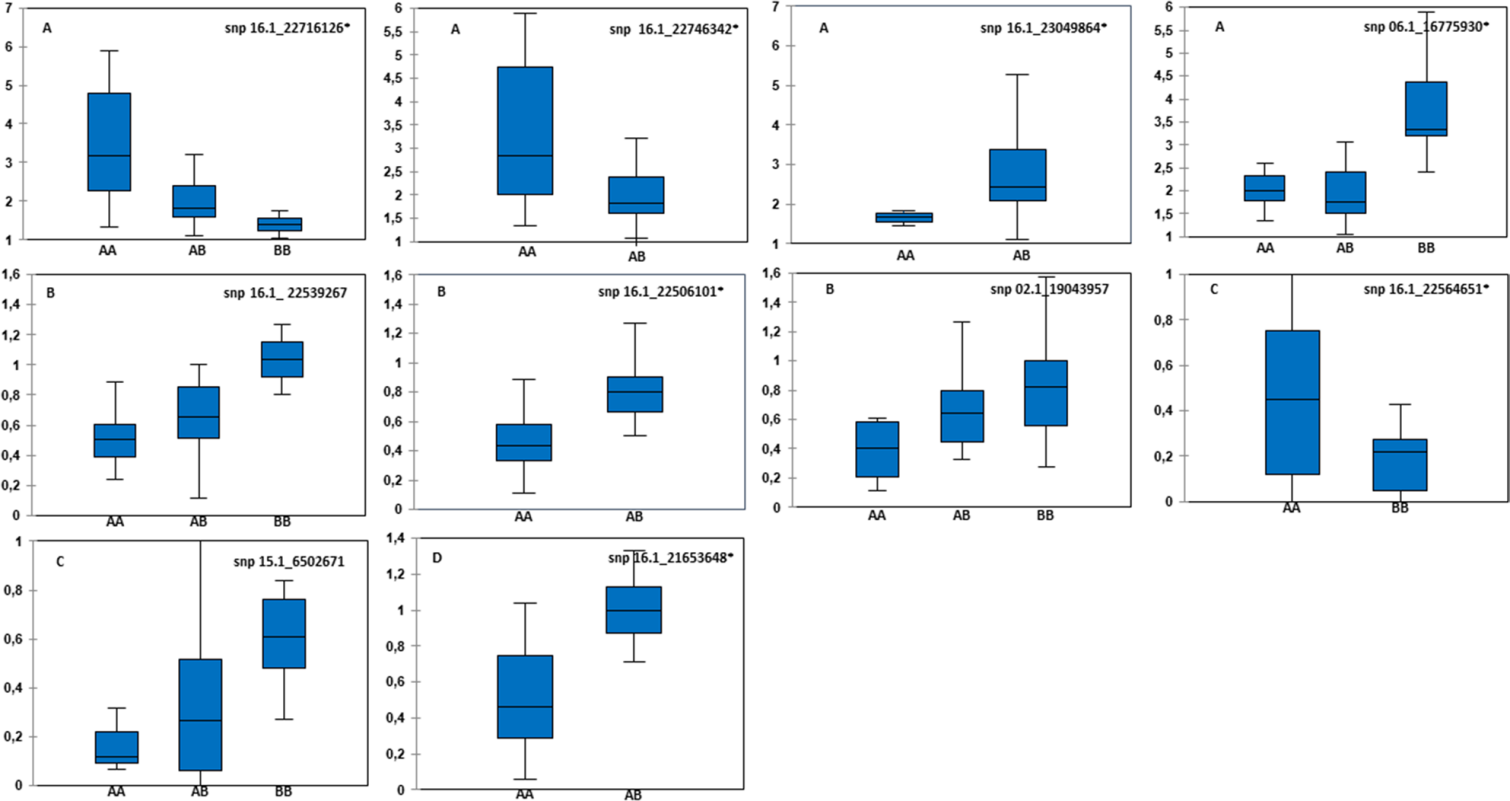
Box plots of the distribution of phenotypic data in the validation panel according to the observed genotypic classes. (**A**) Alleles controlling the ratio length/width, (**B**) Alleles controlling shape regularity, (**C**) Alleles controlling skin texture, (**D**) Alleles controlling tubercular roots. The letter A refers to the reference allele (when calling for variants on the *D. rotundata* genome) and B to the alternate allele. *The observed genotypic classes were significant different at p < 0.05.

Figure 4 shows the distribution of length/width ratio values according to genotypic classes for the SNP 16.1_22716126 marker. Three different genotypic classes were observed in the panel at this locus (AA, AB BB). The letter A refers to the reference allele (when calling for variants on the *D. rotundata* genome) and B is the alternate allele. Allele B reduced the length/width ratio and was transmitted by the 74F female and Kabusa male, both of which were heterozygotes.

Three QTLs were validated for tuber shape regularity (Table 6, Fig. 4) i.e. SNPs 16.1_22539267 (R^2^ = 0.154, p < 0.047), 16.1_22506101 (R^2^ = 0.376, p < 0.001) and 02.1_19043957 (R^2^ = 0.178, p < 0.032).

Two QTLs were validated for skin texture one on chromosome 16, the SNP 16.1_22564651 (R^2^ = 0,169 p < 0.04144.9] and the second on chromosome 15 i.e. SNP 15.1_6502671 (R^2^ = 0.171, p < 0.036]). At this locus, three genotypic classes were observed in the diversity panel (AA, AB, BB). Allele A was responsible for smooth skin (Fig. 4). This allele was transmitted by Kabusa male (AB) and was absent in the rough skinned female (BB).

Finally, a QTL was validated for tubercular roots i.e. SNP 16.1_21653648 (R^2^ = 0.182, p < 0.033).

The analyses of variance showed that differences between the observed genotypic classes were significant (*P < 0.05, Fig. 4) for the majority of validated SNPs markers.

### Identification of potential candidate genes

Putative genes located around the validated SNPs were identified by searching in the NCBI database, which contains all 35,078 genes that were anotated on Dioscorea rotunda reference genome^37^.

We found two candidate genes for trait tuber size, one near SNP 16.1_23694121 (a cell division cycle 202 gene, a cofactor of the APC complex) and a second near SNPs 16.1_22716126 and 16.1_22746342 (a cell number regulator 6 gene). The APC complex is an E3 ubiquitin ligase and a critical regulator of cell cycle progression^38^. While the family of CNR (cell number regulator) genes is known to affect plant and organ size^39^.

Regarding the presence of tuberous roots, a candidate gene was found near SNP 16.1_21653548 (a Squamosa promoter binding like protein 8 gene). SQUAMOSA Promoter-binding protein-Like (SPL) affect a wide range of biological processes, including leaf initiation rate, trichome production, anthocyanin and carotenoid biosynthesis and lateral root development^40^.

## Discussion

### Inheritance of tuber quality traits

The analyses of variance and broad heritabilities calculated for each trait revealed that genetic factors involved in the expression of different tuber quality characters were very important.

Tuber shape regularity heritability has been reported to be moderate (0.58) or high (0.845) in potatoes depending on the studies^25,41^. In our study, the heritabilities in both biparental populations were significantly different. Heritability was found to be moderate in population A (0.55), while it was higher in population B (0.82). This difference could be explained by the fact that the genotypes in population A had more regular forms with less digitation than those in population B. In other words, there was less variability in population A for this trait than in population B.

Similarly, a significant difference in heritability values was observed for tubercular roots. Heritability was lower in population A, (0.34) than in population B, which was moderate (0.51). This lower heritability for population A could be explained by the very low variability observed in this population for this trait.

The presence of transgressive hybrids (with phenotypic values higher than those of both parents) in the B population for tubercular roots and shape regularity traits could be due to the heterozygosity of both parents. Indeed, both parents of this population (74F and 14M) had a very high heterozygosity rate^19^ which would result in new allelic combinations created at the locus concerned, thus widening the range of phenotypic variation within the population.

Heritabilities for the tuber shape, skin texture, flesh colour and tuber flesh oxidation traits were high and almost identical in both populations. In potato, tuber shape heritability was reported to range from 0.64 to 0.90 depending on variability present in the studied populations and on the extent of differences between years and environmental effects^14,25,42^.

### QTL detection

A total of 34 QTLs associated with quality traits were detected and were distributed on eight different chromosomes (LG01, LG02, LG06-M, LG07, LG15, LG16, LG17 and LG19). However, more than half (62%) were positioned on the LG16 chromosome, indicating that this chromosome likely plays an important role in the genetic determinism of the traits studied. Indeed, QTLs were detected in three different regions on this chromosome. However, QTLs in the lower part of the chromosome were only identified in population B. In addition, more QTLs were identified in population B than in population A. This could have been due to the larger size of this population^43^, as well as to the greater variability observed for some traits during phenotyping. This high number of QTLs detected in the two populations revealed the complexity of the characters studied.

Breeding for tuber shape regularity is a long and difficult process because this trait is markedly affected by environmental factors and especially by the soil structure^44^. Access to markers associated with genes controlling this trait would thus greatly enhance the speed and efficiency of the selection process.

QTL analyses on population B led to the identification of five important loci for tuber shape regularity, while explaining 90% of the total phenotypic variance. These results showed that tuber shape regularity was a polygenic trait, which is consistent with previous results reported in potato^25,41^. Seven QTLs were identified by Hara-Skrypiec et al.^25^ in diploid progeny generated from genitors having several *Solanum* species in their pedigree (*S. tuberosum*, *S. acaule*, etc.), whereas four QTLs were detected in tetraploid *Solanum tuberosum* progeny by Bradshaw et al^41^. No QTLs influencing this trait were identified in population A, thereby highlighting the interest of using highly heterozygous genitors (hk × hk) to detect QTLs for tuber shape regularity. All of the QTLs actually showed 1:2:1 segregation. For most loci, the phenotypic values obtained for heterozygous genotypes were intermediaries to that of homozygous, suggesting partial dominance of genes controlling this trait. The presence of genotype carriers of alleles increasing tuber shape irregularity in the homozygous state was probably responsible for the greater diversity observed in population B (transgressive hybrids having highly malformed tubers), which allowed identification of QTLs.

Three QTLs were validated for tuber shape regularity in the diversity panel, which showed that the revealed loci were not specific to the genitors’ genetic background. For example, in the case of SNP 02.1_19043957, three different genotypic classes were observed in the panel. Varieties known to have a very regular shape were homozygous for the favourable allele, while the other varieties were either homozygous or heterozygous for the allele increasing tuber irregularity. The validated QTLs should thus be very useful for selecting the best parental combinations and promising hybrids in segregating populations.

Tuber shape is another important trait and is known to be highly variable in *D. alata*^45^. The shape can be cylindrical (with more or less elongated tubers), flattened, oval or round. This characteristic was quantitatively assessed based on the length/width ratio. As the 74F female parent produced very long tubers (mean ratio = 9.54) compared to 14M and Kabusa males (2.09 and 1.52, respectively), high variability was observed in both progenies. A total of five QTLs were identified on three different chromosomes, accounting for 64% and 66% of the phenotypic variation observed in populations A and B. Our results thus revealed that the length/width ratio is a complex genetic trait. Similar results were obtained in potato, where 7 significantly different loci were detected for tuber shape^14,25,41,42^. In potato, progeny phenotyping was carried by two different approaches using a 1–4 breeder scale (1 = round, 2 = oval, 3 = long oval, 4 = very long oval) and the length/width ratio. In addition, the number of QTLs revealed was dependent on both the genetic background of the populations and the phenotyping and QTL detection methods implemented. Using the composite interval mapping (CIM) approach, Prashar et al.^14^ detected additional QTLs compared to those identified using simple interval mapping (SIM). In our case, the CIM approach did not allow the detection of additional QTLs. Three QTLs were validated in the diversity panel for tuber shape, two of which had a positive effect and one a negative effect on the length/width ratio. The validated markers should thus be useful for selecting alleles that reduce the length/width ratio to generate hybrids with medium-sized tubers.

Presence of roots on tuber surface make the tubers unattractive and also peeling difficult during preparation. A high variability exists in D. alata for this trait. Some cultivars present a large number of roots while others have few or no tubercular roots^46–48^. Five QTLs associated to the presence of tubercular roots were identified on population B accounting for 91% of total phenotypic variance. One QTL that explained 20% of phenotypic variance was validated on the diversity panel. This had a 1:2:1 segregation in population B. The phenotypic values of heterozygous genotypes were intermediaries to that of homozygous, showing partial dominance of gene controlling this trait. Allele increasing the number of tubercular roots was not observed at homozygous state in the diversity panel, suggesting that this allelic configuration has probably been contra-selected.

The skin surface of tubers of yam cultivars can be smooth or rough. Tubers with a rough skin surface are more difficult to peel than those with a smooth skin, which can lead to a significant peeling loss. Two QTLs were validated for skin texture on the diversity panel. Locus mapped on chromosome LG15 had a 1:1 segregation and was transmitted by the Kabusa male. Homozygous genotypes had a rough skin as genitor female while heterozygous genotypes had a smooth skin like genitor Kabusa, showing that allele conferring a smooth skin is dominant.

QTLs associated to flesh colour and oxidative browning of flesh were also identified on both populations. Lowest number of QTLs was detected for flesh colour, suggesting that a few numbers of loci, as showed in potato controls this trait^25^. Bonierbale et al.^49^ identified in potato a major QTL associated to yellow colour. Brown et al.^50^ showed that yellow colour is determinate by carotenoid content and could identify a candidate gene (beta-carotene hydroxylase). In our biparental populations, the flesh colour ranged from white, via cream to yellow. The QTL explaining the higher phenotypic variance (40%) had a 1:2:1 segregation. The phenotypic values of heterozygous genotypes were significantly intermediaries to that of homozygous suggesting partial dominance of gene controlling this trait. However, as we could not make destructive measurements on our diversity panel, QTLs associated to flesh colour and flesh oxidation could not be validated. This would have to be accomplished in order to be able to use them for marker-assisted selection.

The identification of these QTLs as well as some putative candidate genes will be useful to move towards the identification of key genes to better understand the molecular basis of these important morphological and agronomic tuber quality traits.

### QTL colocalization and yam improvement implications

In this study, several QTL colocalizations were found on the LG16 chromosome for many of the studied traits. Moreover, QTL colocalization was observed for the tuber length/width ratio and skin texture on chromosome LG15. The QTL colocalizations confirmed the genetic correlations observed between traits. Indeed, the length/width ratio was strongly and positively correlated with the tuber skin texture, suggesting that both traits could be under control of the same genes. In potatoes, significant genetic correlations were also previously found between tuber shape and shape regularity and between shape regularity and flesh colour, as well as QTL colocalisation^25,51^. These colocations suggest that these traits were dependent on one or more genetically related QTLs, as described in potato by Meijer et al.^52^ and Hara-Skrypiec et al.^25^. The studied traits could therefore be either under the control of several distinct and closely related genes, or of a single gene with a pleiotropic effect. Both hypotheses seem plausible since the genetic correlations were more or less strong depending on the traits. Furthermore, we found that correlations between traits in population B were generally stronger than in population A, which could be explained by the different alleles transmitted by male genitors since the female genitor is the same. In addition, we found that most of the correlations involving the shape regularity and tubercular roots traits were not significant in population A while they were highly significant in population B, which could also be explained by the alleles inherited from male genitors at locus controlling these traits.

The colocalization of quality-related QTLs is a major phenomenon that could have an impact on genetic improvement. The allele associated with QTLs could lead to the improvement of one of the two characteristics while being unfavourable for the second characteristic and therefore could not be used for yam improvement^51^. If an allele is favourable for both characteristics, then that region is of interest for the simultaneous improvement of both characteristics. In our study, QTLs controlling the length/width ratio colocated with those of tuber shape regularity, flesh colour and skin texture and tubercular roots. This implies that markers associated with QTLs could be used for assisted selection in yam breeding and selection. In tangible terms, this highlights the benefits of using elite progenitors while combining many traits of interest to facilitate transmission of target characteristics to progeny, e.g. this is the case for cv Kabusa, which produces medium-sized tubers with a regular shape, no tuberous roots, smooth skin and white flesh.

### First evidence of paracentric inversion in yam

Comparative parental map analyses revealed that the Kabusa male had an inversion of about 280 Kb in chromosome 16. An inversion is a structural chromosomal rearrangement involving two breaks and reinsertion of the chromosome segment after a 180° rotation, thereby reversing the gene order^53^. Our results showed that it was a paracentric inversion because it did not involve the centromeric region. Paracentric inversion is one of the most common forms of chromosome polymorphism found in nature^54^. These inversions are more widespread than translocations and pericentric inversion because they are less likely to produce chromosomally unbalanced offspring^53^. Paracentric inversions play an important role in the speciation of several plant families such as Solanaceae^55^ and Poaceae^56^. Intraspecific variability has been reported in many crops such as cotton^57^ and grapevine^58^.

Carriers of paracentric inversions generate viable gametes (50% normal, 50% inverted) if there is no crossover in the inverted segment, since there is no missing genetic information. On the contrary, if the genetic recombination phenomenon occurs in the inverted fragment, four segregational products are formed (normal chromatid, inverted chromatid, dicentric chromatid, acentric fragment). The two latter very seldom give rise to a viable zygote because they are unstable during mitotic division^59^.

When we produced both populations, major differences were observed in the ratio of generated embryos to the number of pollinations between population A (140/250 = 56%) and population B (280/360 = 78%). The lower rate in population A was related to gametophyte selection^19^ and may have been the consequence of the chromosomal inversion revealed in the Kabusa male. In addition, pollen fertility analyses carried out by staining the pollen with Alexander blue also showed that Kabusa fertility was lower than that of the 14M male (40% and 80% respectively, unpublished data). A reduction of pollen viability caused by inversions was previously reported in several species^60,61^.

As paracentric inversions reduce the recombination rate^55^, we compared recombination rates of both males in the inversion region. The results confirmed that the recombination level in the Kabusa male was significantly lower (14%) than in the 14M male (30%). The value of the latter was congruent with the fragment size. Their fertility can be studied in detail since some recombinants were generated. Moreover, the Kabusa male could also present inversions on other chromosomes and we intend to verify this through an exhaustive comparative analysis of the parental maps of all the chromosomes. Recently, we detected highly putative structural variations on chromosome 6, which is involved in the sex determinism (male = XY and female = XX) ^19,62^. The latter study revealed significant differences in a region of about 3 Mb between chromosomes Y and X (probably related to structural variations).

*Dioscorea alata* breeding programs are hampered by the low fertility of many cultivars. It is possible that structural variations (paracentric and pericentric inversions or translocations) are the cause of this low fertility. New genomic approaches and tools could be useful for investigating the prevalence and evolutionary role of structural variations in* D. alata*.

## Data Availability

The reference genetic map used was described in Cormier et al.^19^. The Illumina HiSeq 3000 sequencing raw data are available in the NCBI SRA (Sequence Read Archive), under the BioProject number: PRJNA515897. The phenotyping datasets are available in Yambase Plateform (https://yambase.org) and from the corresponding author on reasonable request.
